# Research on the ditching resistance reduction of self-excited vibrations ditching device based on MBD-DEM coupling simulation

**DOI:** 10.3389/fpls.2024.1372585

**Published:** 2024-04-08

**Authors:** Ye Zeng, Jun Li, Hongcai Li, Qianqian Zhang, Can Li, Zhao Li, Runpeng Jiang, Chaodong Mai, Zhe Ma, Hongwei He

**Affiliations:** ^1^College of Engineering, South China Agricultural University, Guangzhou, China; ^2^Guangdong Laboratory for Lingnan Modern Agriculture, Guangzhou, China; ^3^National Key Laboratory of Agricultural Equipment Technology, Beijing, China

**Keywords:** self-excited vibration, ditching resistance, ditching device, coupling simulation, bench test

## Abstract

In plant horticulture, furrow fertilizing is a common method to promote plant nutrient absorption and to effectively avoid fertilizer waste. Considering the high resistance caused by soil compaction in southern orchards, an energy-saving ditching device was proposed. A standard ditching blade with self-excited vibration device was designed, and operated in sandy clay with a tillage depth of 30cm. To conduct self-excited vibration ditching experiments, a simulation model of the interaction between soil and the ditching mechanism was established by coupling the ADAMS and EDEM software. To begin with, the ditching device model was first set up, taking into account its motion and morphological characteristics. Then, the MBD-DEM coupling method was employed to investigate the interaction mechanism and the effect of ditching between the soil particles and the ditching blade. Afterwards, the time-domain and frequency-domain characteristics of vibration signals during the ditching process were analyzed using the fast fourier transform (FFT) method, and the energy distribution characteristics were extracted using power spectral density (PSD). The experimental results revealed that the vibrations ditching device has reciprocating displacement in the Dx direction and torsional displacements in the θy and θz directions during operation, verifying the correctness of the coupling simulation and the effectiveness of vibrations ditching resistance reduction. Also, a load vibrations ditching bench test was conducted, and the results demonstrated that the self-excited vibrations ditching device, compared with common ditching device, achieved a reduction in ditching resistance of up to 12.3%. The reasonable parameters of spring stiffness, spring damping, and spring quality in self-excited vibrations ditching device can achieve a satisfied ditching performance with relatively low torque consumption at an appropriate speed.

## 1 Introduction

Ditching is an important agricultural practice used to apply fertilizers. Optimizing the ditching device can not only crush and mix soil but also provide better growth conditions for crops (Min et al., 2021). Thus, crop yields can be effectively increased. It offers advantages such as strong adaptability and good soil crushing performance (Du et al., 2022). At present, there are several main problems in the operation of existing ditching machines, such as a high ditching resistance, high power consumption, poor adaptability of the ditching tools, unstable quality of the ditching operations, and susceptibility to the influence of the geological environment. To solve these problems, researchers have mainly focused on optimizing the bionic structure or working parameters of ditching tools. Wang et al. (2020) designed a new type of ditching knife by bio-mimetic modeling of the ditching blade based on the behavior of moles performing soil excavations, which model encounters less resistance during operation. Sun et al. (2020b) designed a new type of low-resistance ditching tool by imitating the brown bear claw and optimized it through response surface analysis to obtain a dual multivariate regression model based on red soil ditching parameters, verifying the low-resistance characteristics of the designed bio-mimetic ditching tool. However, current design of soil bio-mimetic forms by scholars has difficulty in meeting higher requirements with the increasing precision and efficiency requirements of agricultural machinery (Li et al., 2016; Liu G. et al., 2022). Considering parameter optimization during operation, it is necessary not only to consider single factors but also to consider coordination with other factors. Based on the aforementioned profiling research, Zhao et al. (2021) proposed a bio-mimetic tool according to the biological characteristics of rabbit claws, toes, and nails and added a self-excited vibration deep loosening device, which significantly reduced the torque compared to traditional ditching tools. Research has also shown that introducing vibration into the loosening machine can effectively reduce energy consumption during excavation and soil breaking. Compared with various methods for reducing resistance through excavation and soil breaking, vibration-type ditching and soil breaking have the best effect on reducing resistance (Miah et al., 2016). The vibration methods are mainly divided into forced oscillation method (FOMs) and self-oscillation methods (SOM). FOM is generally achieved by driving oscillation components through the motor shaft, while the excitation source of SOM come from external forces such as soil resistance (Wang X. Z. et al., 2023). Although FOM can be used to reduce the tillage capacity, additional mechanisms are needed, and certain limitations still exist. The SOM mechanism has the same drag reduction effect as the FOM mechanism but does not require an additional excitation transmission system, thereby avoiding additional energy consumption (Cui et al., 2016; Zhao et al., 2021). Bai et al. (2016) investigated the performance of a badger claws bionic subsoiler in reducing tillage resistance, and results showed that the tillage resistance was reduced by 13.05% to 18.94% compared to the tillage method without vibration. Therefore, setting vibration parameters reasonably to control soil disturbance and reduce power consumption has become a research topic that requires further investigation.

Simulation technology provides an efficient and feasible method for studying the soil disturbance process in deep loosening, which can effectively reveal the impact of deep loosening machines on soil trenching fragmentation (Dong et al., 2022). Regarding the simulation research on ditching equipment, the dynamic simulation of equipment and the simulation of soil particles are mainly focused on. To simulate the movement behavior of loose particles such as soil, the Discrete Element Method (DEM) can be used to calculate the behavior state of each particle and investigate their interactions with adjacent particles and geometry (Xiong et al., 2018). With the continuous improvement of computing power, this method becomes applicable to large-scale simulation problems only in recent days. Wang J. W. et al., (2023) conducted a three-dimensional simulation experiment on the interaction between straw-soil trenching machine using the DEM. Through simulation analysis, the study obtained the resistance variation patterns of the ditcher operation. Zhao et al., (2021) designed a deep loosening shovel and used discrete element software EDEM to simulate and analyze the effect of shovel on soil in response to the problem of high soil disturbance and tillage resistance in deep loosening operations in Northeast China. After experimental analysis, the deep loosening shovel effectively reduced soil disturbance and tillage resistance. By comparison, ADAMS was used to simulate complex device motion behaviors, numerically simulating a system composed of multiple objects with consideration of the mass, inertia, and degrees of freedom (Wang J. W. et al., 2023), and has been proven to be an effective tool for motion analysis of multi body dynamics (MBD). Some researchers (Yuan and Yu, 2020; Kim et al., 2022) found that the coupling algorithm of DEM and MBD can be used to simulate and analyze the process of complex device operation, such as the vibration of springs. By utilizing the simulation process of the equipment and the interaction with soil particles, the resistance reduction mechanism of the machine can be qualitatively explained. A study from Liu W. et al. (2022) showed that a discrete element flexible model of tiller taro harvesting, which can be composed of particle mechanics system and MBD method, conducting the clamping and pulling experiments. The purpose of investigating the vibration ditching by coupling simulation is to develop innovative techniques that effectively decrease the resistance during the ditching process, while minimizing the power consumption required for tillage operations (Shao et al., 2019). Ani et al. (2018) stated that the two or more methods can be combined to model the interaction between soil and machinery.

In this study, considering the influence of vibration characteristics on ditching resistance, a self-excited vibration ditching device was designed and simulated to ensure the performance of ditching. The power spectrum analysis was conducted on the vibration signal, evaluating the vibration displacement and vibration frequency characteristics of the ditching blade. The parameters of the self-excited vibration device were optimized and analyzed using the single factor analysis method. In addition, the MBD-DEM coupling method was used to investigate the interaction mechanism between ditching blades and soil particles during the process of deep ditching. The accuracy of the coupling model and the resistance reduction effect of self-excited vibration ditching blades were verified through bench tests and theoretical methods comparison. This approach will help to improve the efficiency of soil tillage in modern orchard production scene and reduce energy consumption, which is of significant importance for energy conservation and cost reduction.

## 2 Materials and methods

### 2.1 The mechanism of vibration ditching

**Figure 1:**
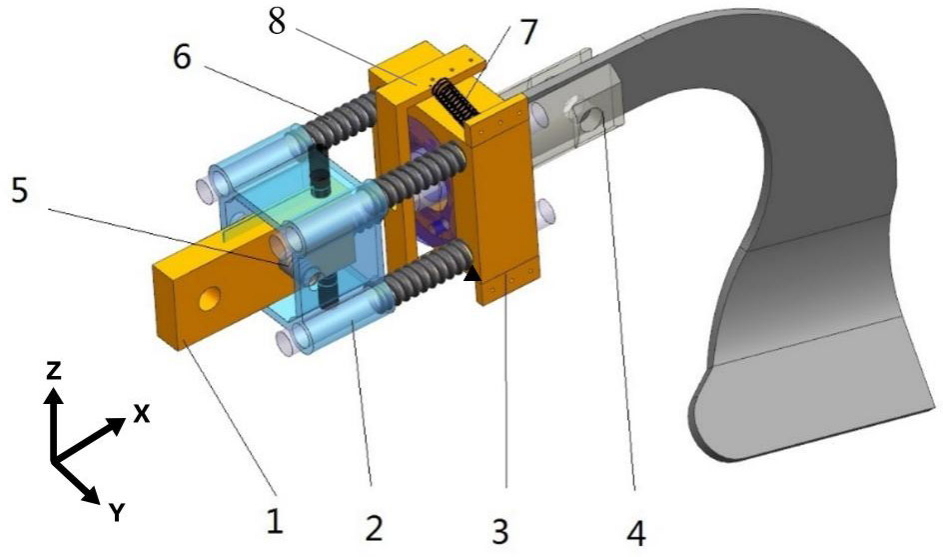
Schematic diagram of the self-excited vibrations device. 1. Bracket; 2. Square mounting seat; 3. Bearing seat; 4. Rotating blade shaft; 5. Spring Z,; 6. Spring X,; 7. Spring Y,; 8. Torsion beam.

During the deep ditching process, due to uneven soil surfaces, changes in the tillage depth and the influence of soil moisture content can affect in the tillage resistance (Bhuiyan et al., 2023). Yang et al. (2024) proposed that during the period of 2015-2020, under conventional tillage, the average soil moisture content in dryland area at depths of 0-60cm was between 16-20%. When the soil moisture is below 20%, soil compaction will happen, which is not conducive to plant growth. Hu et al. (2024) revealed that deep tillage can reduce soil compaction, which has a positive impact on crop root growth and nutrient acquisition. The selection of ditching device is one of the important parts of deep tillage operations. The traditional ditching devices will instantly generate significant resistance when encountering obstacles (such as stones) during the working operation, and exacerbating tool wear that affects the lifespan of the blades and increases energy consumption (Yang et al., 2019). The research and application of existing vibration ditchers have shown that appropriate vibration frequencies and amplitudes can reduce soil tillage resistance and improve soil tillage quality. Therefore, an innovative self-excited vibrations ditching device has been designed, comprising a bracket, a bearing seat, a rotating blade shaft, springs, and a torsion beam, as shown in **Figure 1**.

The bracket is installed on the upper end of the blade and the pressure springs are installed on the bracket along the X, Y, and Z directions, which can release the degrees of freedom of the blade in three directions. The device can achieved self-excited vibration through the elasticity within a specific range. For different soil conditions, the stiffness of the corresponding degrees of freedom can be adjusted to ensure the effectiveness of the operation. The vibrations device is connected to the blade through the rotating blade shaft, which generates three degrees of freedom vibrations to the blade. During the tillage process, affected by external forces, friction occurs between the machinery and the soil. The spring vibration energy generated by friction leads to an increase in the internal stress of soil. With the increase of internal stress, the blade undergoes an uneven self-excited force, resulting in a small amplitude self-excited vibration, thus the compacted soil is more easily crushed by machinery (Li et al., 2020). The vibrations blade can improve the structure of the soil, allowing the root system to extend and grow more easily. And vibration ditching can quickly and accurately form well-distributed grooves, providing an ideal tool for secondary tillage operation. Tillage operations mainly rely on the spring of the device, which undergoes elastic deformation under the action of soil ditching resistance, leading to self-excited vibrations. When the ditching blade is not subjected to force after excavation, the spring elastic deformation is restored. Under the appropriate vibration amplitude and frequency conditions, the average torque can be effectively reduced, achieving the goal of reducing resistance and consumption. Due to the uneven soil structure, the blade can adaptively adjust the relative angle and orientation with the cutter head within a certain range, avoiding the most solid position of the soil and achieving a sliding ditching effect, thereby resulting in interactions with less ditching resistance. Especially the blade is capable of generating adaptive deflection and contraction when encountering hard soil or rocks, thereby reducing wear and tear, as well as minimizing input torque.

### 2.2 Kinematic analysis of the vibrating ditching machine

The ditching equipment used in this study is mainly used in orchards and fields, which have high soil hardness and require a moderate soil throwing distance. In previous design studies, standard grooving curved knives were often used, and the blade design of curved blades were mainly based on the side ditching edge and the tangent ditching edge. There are types of curves on a curved blade, such as helical curves (Archimedean curves) and sine exponential curves. The trajectory curve of the blade is a tangent spatial curve, locating on the cylindrical surface of the rotating blade shaft, which is a circular arc projected on the side view. The ditching trajectory of a traditional ditching machine is a composite motion based on the forward speed and the angular velocity of the ditching blade. The working state of the ditching machine is divided into forward and reverse rotation. Forward rotation refers to the direction opposite to the forward speed of the ditching machine after the blade ditch the soil, while the reverse horizontal speed is consistent with the forward direction. Usually, forward rotation is suitable for excavating wider and deeper ditches, while reverse rotation is more suitable for quickly filling shallow ditches or pits. When rotating forward, the blade applies downward pressure to cut the soil and push it back. When reversing, the soil ditching process moves from bottom to top toward the unconstrained area (surface), pushing the soil forward under the action of ditching tension. By taking the position of the blade shaft axis at a certain moment as the origin, the forward direction of the unit is determined to be the X-axis positive direction, and the vertical upward direction is determined to be the Z-axis positive direction. Thus, the motion trajectory of the blade endpoint can be established as shown in the following equations:


(1) x=vt+r cos ωt

(2) z=−r sin ωt

The ratio of the rotation tangent speed at the tip of the blade to the forward speed of the ditching machine is defined as the ditching speed ratio γ in Equation 3:


(3) $$\text{γ}=\frac{v_{b}}{v}=\frac{r\text{ω}}{v}$$

where r is the rotation radius of the blade, t is the ditching time, v is the forward speed, ${\text{v}}_{\text{b}}$ is the linear speed of the blade tip, and ω is the angular velocity of the ditching blade.

The above equation indicates that when γ is different, the motion trajectory of the ditcher and the shape of the ditching soil are different, and a wavy protrusion will appear at the bottom after ditching. The height of the groove protrusion is related not only to the number of blades set in the ditching area of the groove cutter’s roller unit and the turning radius of the groove cutter head but also to the groove speed ratio λ Related studies have shown that when the turning radius of the cutter head and the number of blades are constant, a larger grooving speed ratio will result in a smaller protrusion height (Dong et al., 2022). Although the power loss during forward rotation is lower than that during reverse rotation under the same ditching parameters (Ding et al., 2018), the backward throwing of soil during forward rotation can impact the effectiveness of ditching. During reverse rotation, the pitch of soil ditching is larger than that of forward rotation, which can improve the compactness and stability of the soil. To avoid the phenomenon of the back of the blade squeezing the soil in front of it during the ditching process, the ditching depth of the ditching machine, the forward speed, and the linear speed of the tip of blade rotating with the blade shaft must meet the following relationship (Zhang et al., 2013) as Equation 4:


(4) $$\text{γ}=\frac{v_{b}}{v}>\frac{\text{r}}{\text{r}-\text{h}}\;\;$$

where h is the depth of the ditching machine, and h<r .

The soil nutrient requirements for fertilizer management and return cultivation in orchard environments are usually distributed in shallow soil layers, ranging from 0 to 40 cm deep, with 0-15 cm of topsoil and 15-40 cm of subsoil. Excavating ditches within this range can result in a better root system area for plants, providing sufficient growth space and nutrients (Lv et al., 2023). Therefore, controlling the depth of ditching to h<40 cm not only helps plants gain access to more nutrients and water but also helps to reduce the power loss of the ditching machine (Liu et al., 2023). Due to the small turning radius of the cutter shaft, the blade reversal method is used for drag reduction research. Wu et al. (2021) studied the effects of deep fertilization on gas nitrogen loss intensity, fertilizer utilization efficiency, and crop yield in humid and semi-humid region, applying fertilizer at a depth of 8 cm, the decrease in gas nitrogen loss intensity was significant and crop yield significantly increased. However, when the fertilization depth increased to 35 cm, nitrogen loss was reduced, but fertilizer utilization efficiency and crop yield decreased. Based on the above analysis, the ditching depth is set to 30cm. In order to meet the nutrient requirements of fruit tree, flowering, and crop, generally in autumn, ditching and fertilization operations are carried out directly below the outer edge of the tree crown. According to horticulture requirements, the width of the ditching should be between 10-30 cm (Wang J. W. et al., 2023). This experiment selected a ditch width of 15 cm for testing. Considering the small amplitude vibration of the blade during the working process and according to Equations 1 and 2 of the traditional blade, the trajectory of the vibrating blade can be determined as shown in Equations 5 and 6:


(5) x=vt+(r+s(t)) cos ωt

(6) z=−(r+s(t)) sin ωt

where s(t) is the vibration amplitude, with the direction along the blade axis.

The introduction of a time-varying vibration amplitude leads to changes in the blade turning radius over time, thereby affecting the changes in the tool tip trajectory and speed during the turning process. Due to the presence of multiple frequency components in the vibration during blade-soil contact, this amplitude is expressed through harmonic polynomials. Generally, the amplitude of low-order harmonics is larger, and the frequency is lower, while the opposite is true for high-order harmonics. The phase is determined based on the angle at which the blade contacts the soil and begins to deform.


(7) $$\text{s}\left(\text{t}\right)={\text{a}}_{1}(\text{sin }{\text{ω}}_{1}\text{t}+{\text{φ}}_{1})+{\text{a}}_{2}(\text{sin }{\text{ω}}_{2}\text{t}+{\text{φ}}_{2})+{\text{a}}_{3}(\text{sin }{\text{ω}}_{3}\text{t}+{\text{φ}}_{3})$$

where ${\text{a}}_{\text{i}}\;$ , $\omega_{\text{i}}$ and ${\text{φ}}_{\text{i}}$ are the amplitude, frequency, and phase of the i-th harmonic, respectively, and i=1, 2, and 3.

**Figure 2:**
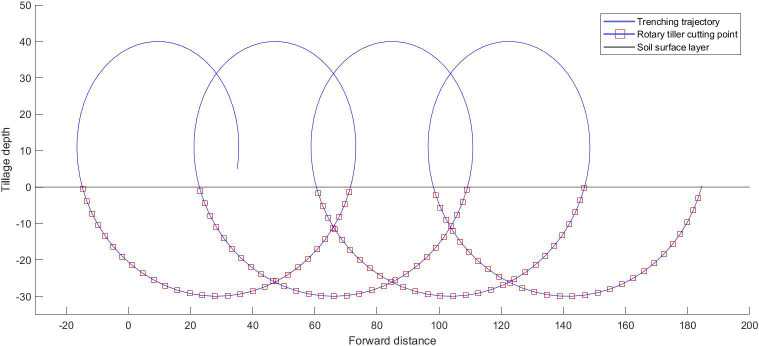
The trajectory of the ditching blade.

By substituting Equation 7 into Equations 5 and 6, the amplitude is found to be generally larger than that of the higher harmonic due to the lower frequency of the first harmonic; thus, i=1. The common soil type in southern China is sandy clay soil, which usually has high viscosity and plasticity. A slower forward speed can reduce resistance and energy consumption, resulting in a relatively good work efficiency. According to the common rotary tillers’ working parameter range, the middle value of the motion parameters is determined to the forward speed of the tool, which is set to 0.3 m/s (Zhang et al., 2024). According to Equation 7, the speed should be greater than 172 r/min. A numerical simulation was conducted in MATLAB with a ditching depth of 30 cm, a forward speed of 30 cm/s, and a rotating speed of 330 rpm. The ditching trajectory is shown in **Figure 2**.

The ditching depth of the vibrating ditcher has slightly decreased under the same simulation conditions mentioned above. Considering the case of high-frequency micro amplitude vibration, the vibration trajectories with multiple harmonic components are superimposed on the original trench cycloid. The vibration trajectory of this characteristic causes periodic changes in the contact force with the soil under the same experimental conditions. The friction and adhesion forces on the side of the tool are greatly reduced (Zhang et al., 2013), thereby affecting the torque. Numerical simulations were conducted at speeds of 220 rpm, 330 rpm, and 440 rpm. Due to the micro-amplitude vibrations of various degrees of freedom, the variation between the device and soil in the ditching angle during the contact process is caused by the rotational speed, thereby affecting the input torque (Bai et al., 2016). However, the change in the input torque at different rotational speeds is related to the ditching force during the soil ditching process and the inertia force of the soil during the soil throwing process, which is a nonlinear relationship influenced by multiple factors. When the soil hardness is uneven, the blade can adjust the ditching angle slightly according to the gradient direction of the lowest soil hardness, thereby avoiding the solid part and reducing the ditching resistance. During the soil throwing process, the swinging and twisting effects of the blade impact the soil, which can increase the crushing effect and shake off the soil, reducing the amount of soil thrown, saving energy, and increasing the soil coverage rate. The vibration of soil breaking increases the fluctuation of the acceleration and velocity, and the instantaneous change in the soil ditching velocity and acceleration helps to improve the crushing efficiency and effect. When the spring stiffness and speed are within a suitable range, the kinetic energy and potential energy stored by the spring can be used to break through the soil in the next cycle.

### 2.3 The simulation test models

#### 2.3.1 Multi-body dynamics simulation model

To verify the vibration mechanics of the ditching device, a MBD simulation model was established using ADAMS software, and vibration ditching characteristics simulation tests were conducted to explore the internal dynamic vibration characteristics of the device. A fully parameterized virtual prototype model was created using the parts library, constraint library, force library, and interactive graphical environment of ADAMS software. After importing the three-dimensional diagram of the ADAMS model into ADAMS software, the constraints of the model were defined based on the actual situation, and material properties were added. Among them, steel is selected as the geometric material type with density of $7800\text{kg}/{\text{m}}^{3}$ , Young’s modulus of $7\times 10^{10}\text{ pa}$ , and Poisson’s ratio of 0.3. According to the motion of degree of freedom, corresponding constraint conditions are set to establish the MBD simulation model of the ditching device. The MBD model employed in this study demonstrates precise simulation capabilities for accurately imitating the rotation of the ditching blade and the vibration characteristics of the spring.

#### 2.3.2 Discrete element simulation model

Due to the large number of experimental groups, it is not suitable to process the grooving blades separately for testing. Therefore, numerical simulation methods are used instead of actual grooving operation tests (Yang et al., 2018). The main numerical simulation methods for soil ditching include the finite element method (FEM) and DEM (Aikins et al., 2021; Wang et al., 2021). Among them, the discrete element method has good adaptability for simulating soil particles (Yang et al., 2018; Shi et al., 2019). Therefore, in this article, the DEM is adopted. Based on the actual size of the soil groove on the bench test platform, the soil groove size will be reduced in the EDEM simulation to 2000 mm×650 mm×700 mm. Considering the characteristics of the EDEM software, appropriately enlarging the soil particle radius to reduce the simulation time will not affect the simulation results. Therefore, the radius of soil particles in the EDEM simulation soil tank was enlarged in this experiment, and spherical soil particles were used. To accurately simulate the movement of soil particles, a total of 90000 particles were generated for particle size distribution. Three different radii of soil particles were set, specifically 2 mm, 3 mm, and 4 mm.

The study is based on the sandy clay soil in South China, where the soil particle simulation in EDEM has a high moisture content and high viscosity (Wang et al., 2018). The analysis of the motion of sandy clay using the ordinary Hertz-Mindlin model is not specific due to its basis on the principle of interaction between soil and soil. To address this limitation, the EDEM simulation utilized the Hertz-Mindlin model in conjunction with the Johnson-Kendall-Roberts (JKR) contact model as the contact model between particles (Johnson et al., 1971; Liu G. et al., 2022; Tang et al., 2023). To quantify the cohesion between particles, the model utilizes the JKR normal elastic contact force (F_JKR_) for calculations in Equation 8.


(8) $$F_{JKR}=\left(\frac{4{\text{E}}^{*}}{3{\text{R}}^{*}}-4\sqrt{\alpha \pi \delta {\text{E}}^{*}}\right){\text{α}}^{3}$$

where, ${\text{F}}_{\text{JKR}}$ is the normal elastic contact force of JKR, δ is the surface tension, E* is the equivalent elastic modulus, α is the tangential overlap, and R* represents the equivalent contact radius.

In the JKR model, the surface energy represents the bonding force between particles, and it significantly affects the particles fluidity. In this case, the soil particles were determined to have a surface energy of 7.91 J/m^2^ (Wu et al., 2017; Alizadeh et al., 2018). This model is suitable for simulating materials that demonstrate substantial bonding and agglomeration between particles as a result of static electricity or moisture (Dou et al., 2022). The Hertz-Mindlin no slip contact model was utilized as the contact model between the particles and device Horabik and Molenda, (2016); Sun et al (2020a). The soil particles and material parameters are calibrated through soil rotation tests (Tang et al., 2023). As shown in **Table 1**, appropriate contact mechanics models and relevant EDEM simulation parameters were determine through the EDEM preprocessor module.

**Table 1:** Material and contact property parameters.

Parameter	Value (Unit)
Soil density	1650 (kg/m^3^)
Soil Poisson’s ratio	0.4
Soil Young’s modulus	6×10^7^ (Pa)
45 Steel density	7800 (kg/m^3^)
45 Steel Poisson’s ratio	0.3
45 Steel Young’s modulus	7×10^10^ (Pa)
Soil−soil recovery coefficient	0.2
Soil−soil static friction coefficient	0.67
Soil−soil rolling friction coefficient	0.03
Soil-45 steel recovery coefficient	0.2
Soil-45 steel static friction coefficient	0.75
Soil-45 steel rolling friction coefficient	0.1

#### 2.3.3 MBD-DEM coupling simulation model

**Figure 3:**
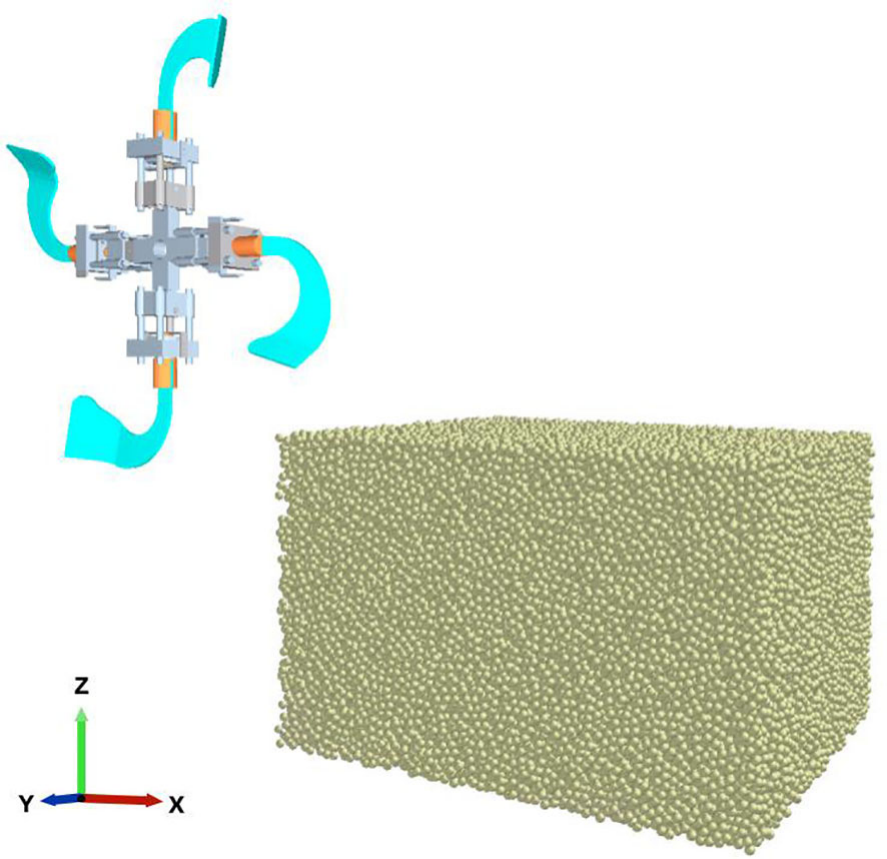
ADAMS-EDEM coupling simulation model.

EDEM cannot define complex mechanism motion processes, such as the spring vibration, which motion module only can achieve simple motion. By comparison, ADAMS can achieve complex movements. The collaborative simulation interfaces and modules enable the transfer of motion information from ADAMS to EDEM to satisfy the simulation requirements of the ditching mechanism (Li et al., 2021; Zhang et al., 2022). To achieve the interaction between the soil and the self-excited vibrations ditching device, the MBD simulation model is coupled with the DEM simulation model. The same model is adopted as the simulation of the ditching components with consistent parameters. In the coupling environment, the positive X-axis represents the orientation of the ditching process. The Y and the Z-axis corresponds to the horizontal direction and the vertical direction respectively. In the post-processing module of ADAMS, the torque, angular velocity, angular acceleration, etc., can be directly exported, and the movement and force situation of soil particles and tillage mechanisms can also be obtained in the post-processing module of EDEM. First, a general force and source settings are added to the moving blades in the ADAMS simulation model. The total simulation time is set to 5 seconds, and a time step of 1000 is used. Then, each motion geometry in the MBD simulation model are imported and coupled in the DEM simulation model. Finally, the interaction between ditching device and soil is achieved through ADAMS-EDEM coupling simulation, as shown in **Figure 3**.

### 2.4 Experimental design

To explore the mechanism of internal load ditching and resistance reduction, a simulation experiment on the load vibration ditching characteristics was conducted, and the simulation results were analyzed. The time-domain and frequency-domain (vibration displacement and frequency) of the three degrees of freedom ( ${\text{D}}_{\text{X}}$ , ${\text{θ}}_{Y}$ and ${\text{θ}}_{\text{Z}}\;$ directions) are determine as evaluation indicators, that vibration characteristics generated by the ditching blade. This indicator is to analyze the influences of various factors, including spring stiffness, spring damping, and blade quality, on the vibration characteristics in the ${\text{D}}_{\text{X}}$ , ${\text{θ}}_{\text{Y}}\;$ and ${\text{θ}}_{\text{Z}}\;$ direction. The obtained vibration curve results are processed using power spectrum. The power spectrum refers to the power spectral density function, which illustrates the variation of signal power with frequency. It essentially represents the distribution of signal power in the frequency domain. By analyzing the power spectrum of the blade vibration data, it is possible to extract the energy distribution of the vibration at different frequency, thereby obtaining the main vibration frequency of the blade. In simulation analysis, the translational degrees of freedom in the X, Y, and Z directions at the center of the MBD simulation model, as well as the rotation degree of freedom in the Y and Z directions, are constrained to release the X-direction degrees of freedom of the tool, which rotates counterclockwise relative to the plane. In the ADAMS-EDEM coupling load ditching analysis, each structural factor is configured with 5 levels, as indicated in **Table 2**. The simulation time is set to 5 seconds, which will be divided into 1000 time steps. In this duration, a total of 15 groups of single-factor coupling simulation tests will be conducted.

**Table 2:** Code table of the structural factors.

Number	Spring stiffness (N/mm)	Spring damping (Ns/mm)	Blade mass (kg)
1	5	0	1.0
2	10	0.03	1.5
3	15	0.3	2.0
4	20	3.0	2.5
5	25	30	3.0

**Figure 4:**
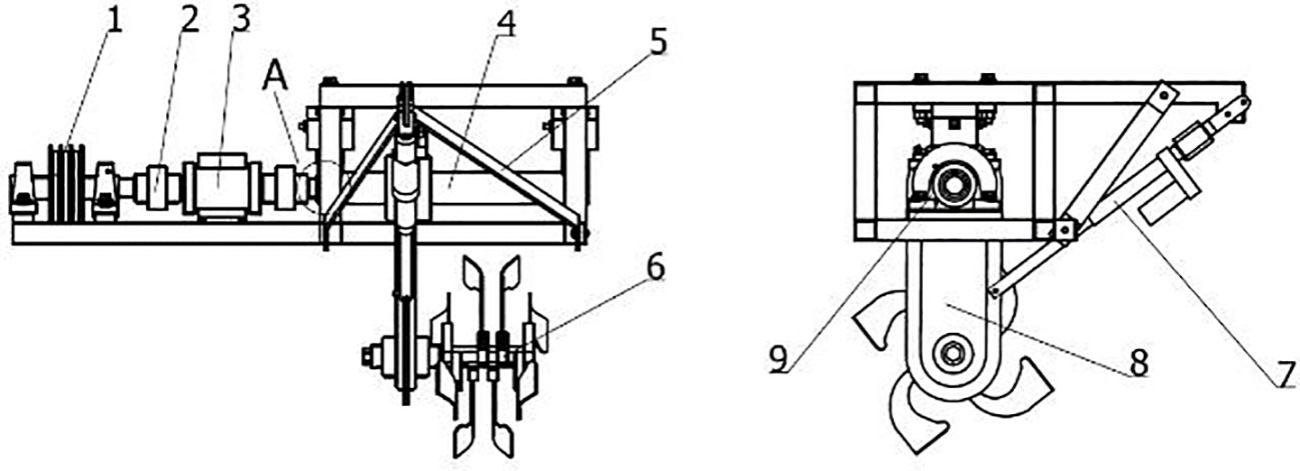
Structure of the bench test device. 1. Drive belt pulley; 2. Coupling machine; 3. Torque and speed sensor; 4. Gearbox support tube; 5. Tillage depth adjustment support frame; 6. Rotating blade; 7. Push rod; 8. Gearbox; 9. Bearing seat.

According to the model of the ditching device and the horticulture requirements, the fixed ditch depth is 30 cm and the fixed ditch width is 15 cm. Based on the analysis of the dynamic vibration characteristics, the device’s rotational speed was set to 330 rpm. The analysis of the formation process of ditching and the mechanism for reducing resistance was conducted, with a forward speed of 30 cm/s. To ensure a more accurate comparison and comprehensive analysis, the ADAMS-EDEM coupling simulation was conducted under identical conditions and with the same working parameters as the bench test. The experiment was conducted on a soil tank laboratory at South China Agricultural University in Guangdong Province. A bench test platform, measuring 9.6 meters in length, 1.5 meters in width, and 1.3 meters in height, was used to analyze the resistance changes during the load ditching process. **Figure 4** illustrates the structure of the bench test device.

A WDH-300Z torque and speed sensor is installed on the experimental platform to detect the torque and speed. The speed of the sensor is primarily determined by the photoelectric switch. As the speed code continuously rotates, the photoelectric switch generates a pulse signal with a specific frequency. By measuring the speed code and the frequency of the output pulse signal, the speed can be accurately determined. The speed of the ditching cutter is controlled using a frequency converter. To evaluate the effectiveness of the coupling simulation and platform testing in terms of ditching, measurements were taken at intervals of 50 cm along the ditching direction. The depth and width of the ditch were recorded under five different speed conditions. Based on the practical operating speed range of the tillage tool, which was determined to be between 180 and 500 rpm, five levels were selected for testing: 180 rpm, 220 rpm, 330 rpm, 440 rpm, and 500 rpm. In order to minimize errors, each experiment was repeated three times to obtain the average values, reducing any random variations and provides more reliable and accurate results. Before each experiment, the sensor was calibrated, the soil moisture was adjusted, and the ditch depth was compacted to maintain the basic consistency of the soil moisture content, firmness, and ditch depth in each experiment (Guan et al., 2022). Using the average resistance of the ditching blade as an evaluation indicator, simulation results are obtained and compared with the variations in average resistances in the bench test.

## 3 Results and discussion

### 3.1 Test results and analysis of load ditching vibration characteristics

**Figure 5:**
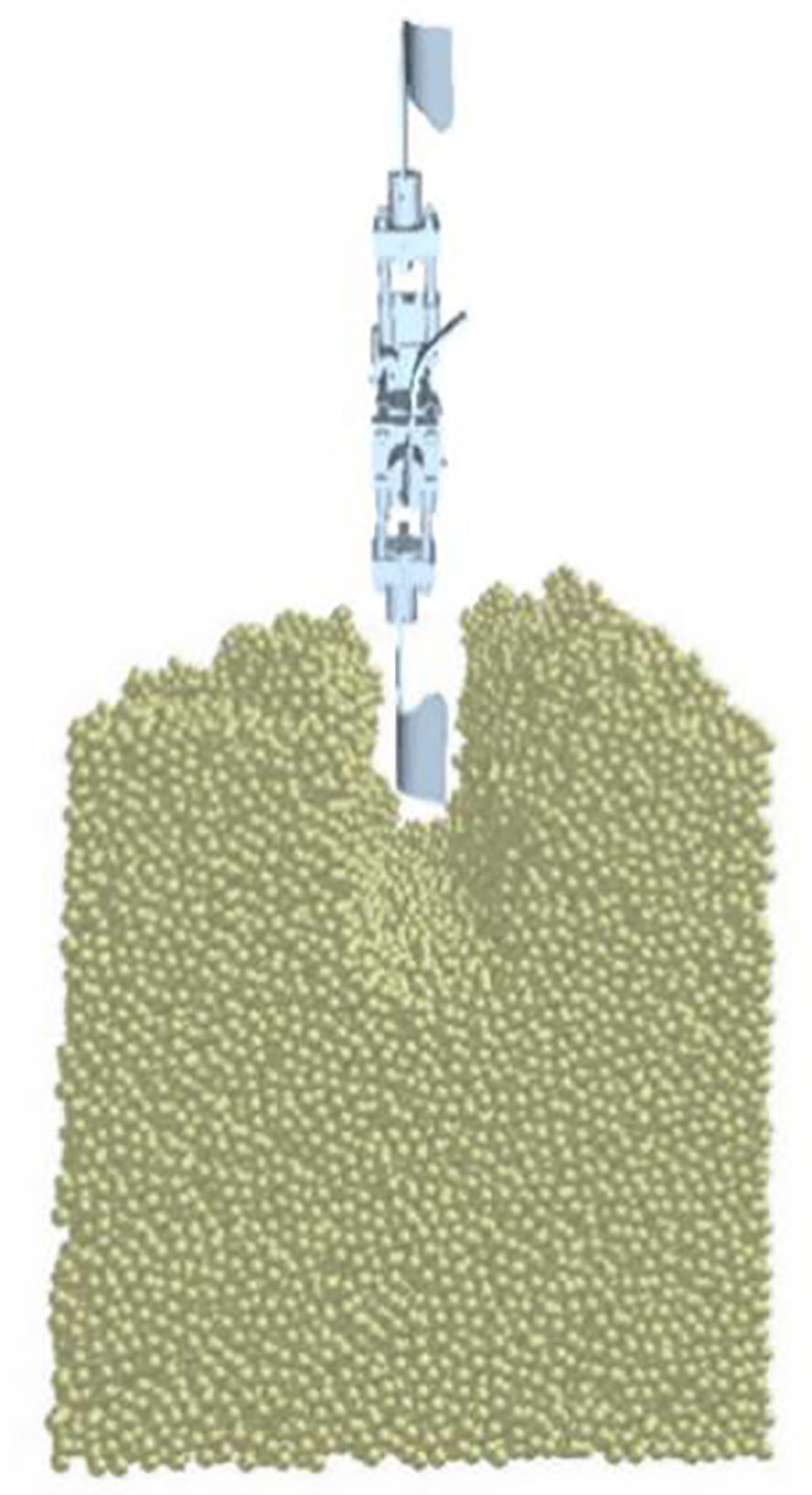
ADAMS-EDEM coupling simulation ditching effect.

By coupling the ADAMS and EDEM software, a load ditching simulation model was established. To investigate the effect of ditching, a ditching experiment was conducted between the vibration blade and the soil tank. The experimental ditching effect are presented in **Figure 5**. Based on the displacement and frequency changes in various directions, the torque variation trends under different conditions were obtained to analyze the resistance reduction and consumption reduction effects of the self-excited vibrations ditching device under different conditions.

#### 3.1.1 The vibration characteristics on the different spring stiffness

**Figure 6:**
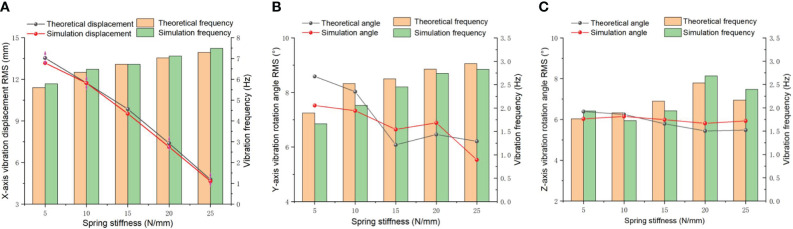
Vibration displacement and variation frequency under different spring stiffness. **(A)** X direction; **(B)** Y direction; **(C)** Z direction.

The ditching blade undergoes adaptive deformation against the resistance when the device is loading ditching, reducing resistance during the ditching operation through spring vibration. The spring stiffness refers to the degree of elastic deformation produced by a spring when subjected to force. The greater the stiffness is, the smaller the elastic deformation of the spring, and the greater the force it bears. The magnitude of stiffness depends on factors such as the material, cross-sectional shape, and length of the spring. Generally, as the stiffness of the spring increases, the pressure it can withstand increases, but it also reduces the elastic deformation and vibration sensitivity of the spring. Therefore, under the conditions of the spring damping of 0.03 N/mm and blade quality of 1.0 kg, the variation of the self-excited vibrations ditching device is analyzed in the time and frequency domains by increasing the spring stiffness, which relationship is presented in **Figure 6A**. The results indicate that the vibration displacement ${\text{D}}_{\text{X}}$ gradually decreases with the spring stiffness increases. The RMS simulation value decreases from 13.172 mm to 4.659 mm. The vibration displacement ${\text{D}}_{\text{X}}$ decreases linearly within the range of 5-25 N/mm of spring stiffness, especially significant decreasing within the range of 10-20 N/mm. The simulation value shows the same trend as the theoretical value, and the vibration displacement decreases with an increasing spring stiffness. The average relative error is 2.29%. According to the power spectrum analysis, it has been observed that the vibration frequency in X-direction gradually increases. The vibration frequency ${\text{f}}_{\text{X}}$ remains between 5.74 and 7.21 Hz, mainly because the centripetal force providing the excitation force that generates the X-direction vibration frequency. The paired t-test using SPSS was used to compare the vibration displacement ${\text{D}}_{\text{X}}$ and vibration frequency ${\text{f}}_{\text{X}}$ of the two sets of data. The calculated paired test results showed that t was 3.229 and 3.52, respectively, based on the degree of freedom of df=4 and significance level of α= 0.05, querying the t-value table to obtain the critical t-value of t_0.05(4)_=2.776. The actual results |t|>t_0.05(4)_ indicate that there is a significant effect on the changes in the spring vibration displacement ${\text{D}}_{\text{X}}$ and vibration frequency ${\text{f}}_{\text{X}}$ in the coupling simulation environment compared to the control. The analysis of the variance (ANOVA) results revealed a highly significant level in the impact of various spring stiffnesses on both the vibration displacement and frequency of the spring (P<0.01).

The relationship between the vibration angular displacement and frequency in the Y and Z directions under different spring stiffnesses is shown in **Figures 6B, C**. The restoring force of the spring raises with the spring stiffness increases, and the Y-direction vibration frequency gradually increases. When the spring stiffness increases from 15 N/mm to 20 N/mm, the vibration angular displacement ${\text{θ}}_{\text{Y}}$ increased to a certain extent. Due to the shortening of the vibration period of the spring, a resonance phenomenon will occur in the amplitude of the spring, which means that the angular displacement of the vibration will slightly increase. However, due to the high energy consumption of the spring in the resonance state, the vibration angular displacement will quickly decrease again when the spring stiffness climbing. The vibration angular displacement in ${\text{θ}}_{\text{Z}}\;$ direction remains within the range of 5.44-6.39°, rare spring stiffness influence. However, the vibration frequency gradually grown from 1.77 Hz to 2.53 Hz. The main purpose of Y-direction and Z-direction vibrations during tillage process is mainly to avoid direct impact between the blade and hard objects, achieving a certain degree of sliding cutting and reducing resistance. It can improve the crushing effect and decrease the proportion of soil throwing. Through ANOVA, it was found that the influence on the vibration angular displacements ${\text{θ}}_{\text{Y}}\;$ and $\;{\text{θ}}_{\text{Z}}\;$ and vibration frequency ${\text{f}}_{\text{Z}}$ did not reach a significant level of 0.05, but the difference in the vibration frequency variation ${\text{f}}_{\text{Y}}$ was very significant (P=0.002). Excessive vibration displacement can increase the ditching resistance to a certain extent when self-excited vibration-ditching blades are operating in soil. When the vibration frequency reaches a certain level, it is beneficial to reduce the friction between the blades and the soil and to quickly shake off the soil attached to the blades through vibration. According to the t-test results, The vibration displacement in three directions is negatively correlated with the spring stiffness. The vibration frequency increases slightly in 5 N/mm-15 N/mm spring stiffness, but the vibration displacement changes is obvious in the X direction. Therefore, the spring stiffness in the X direction should be maintained within the range of 5 N/mm-15 N/mm. During the ditching operation, the spring stiffness should be selected based on the stability and high frequency of the Y and Z vibrations. Therefore, the Y and Z spring stiffnesses should be more suitable at 20 N/mm.

#### 3.1.2 The vibration characteristics on the different spring damping

**Figure 7:**
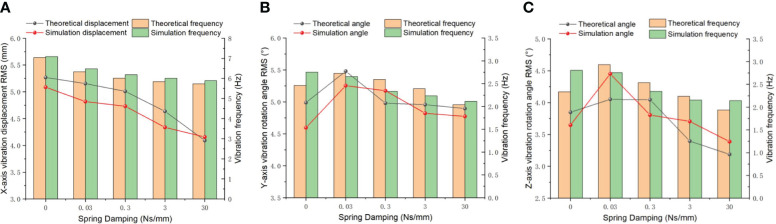
Vibration displacement and variation frequency under different spring damping. **(A)** X direction; **(B)** Y direction; **(C)** Z direction.

Spring damping refers to the inherent damping property exhibited by springs. The magnitude of spring damping is influenced by various factors such as the geometric shape, material composition, and working conditions of the spring. These factors collectively determine the amount of damping present in the spring system. Additionally, the vibration amplitude of ditching machines is affected by these parameters. The larger the damping coefficient is, the smaller the vibration amplitude, but the lower the vibration frequency. Increasing spring damping can effectively reduce the resonance problem present in mechanical structures, thereby further preventing structural damage caused by dynamic stress. When encountering stones during ditching operations, significant external resistance can be absorbed by spring damping, thereby achieving the effect of reducing resistance. In order to ensure reliable vibration reduction during ditching operations, the spring stiffness is set to 10 N/mm. For the Y and Z directions, the spring stiffness is set to 20 N/mm to further enhance vibration. Furthermore, the blade quality is specified as 1.0 kg to ensure accurate simulation results. By adjusting the spring quality, time and frequency domain analysis is conducted on the vibration changes under different spring damping. The vibration displacement and frequency in the X-direction are presented in **Figure 7A**. With the increase in spring damping, the vibration displacement shows a decreasing trend between 0-30 Ns/mm of spring damping, and the overall theoretical displacement RMS decreases from 5.27 mm to 4.092 mm. The simulation value shows a similar trend with the theoretical value. In the simulation analysis, the RMS value fallen from 5.09 mm to 4.16 mm, and the average relative error was 4.26%. According to the power spectrum analysis, both the simulated and theoretical values of the vibration frequency ${\text{f}}_{\text{X}}$ have decreased by more than 1 Hz. A paired t-test can be conducted to compare the vibration displacement and frequency of two sets of data. The calculated paired test results show that t is 2.846 and 6.196, respectively, based on the degrees of freedom of df=4 and significance levels of α= 0.05 and α= 0.01. The t-value table can be referenced to obtain the critical t-values t_0.05(4)_=2.776 and t_0.01(4)=4.604_. According to the actual |t| value, the change in the spring vibration displacement and control in the coupled simulation environment has a significant effect, while the vibration frequency has a very significant effect. The ANOVA indicate that the significant level of the spring damping on the displacement and frequency in X-direction are less than 0.01, which indicates a highly significant effect of spring damping. The RMS value of the vibration angular displacement in Y-direction began to sharply increase and then decreased from 5.48° to 4.90° in **Figure 7B**. The ${\text{θ}}_{\text{Y}}\;$ simulation value showed a similar trend to the theoretical value. In the simulation analysis, the RMS value decreased from 5.25° to 4.77°, with an average relative error of 4.4%. Compared to **Figure 7C**, the vibration angular displacement reduced from 4.05° to 3.19° in Z-direction, especially when the simulated value increased to a certain extent from 3.85° to 4.05° during the 0-0.03 Ns/mm spring damping operation. As the spring damping increases to 0.03 N/mm, the simulated vibration ${\text{θ}}_{\text{Z}}$ angular displacement shows a similar trend to the theoretical value, but there is no significant difference in the changes, with P-values of 0.078 and 0.054, respectively. Through a power spectrum analysis of the angular displacement of Y and Z vibrations in the frequency domain, it is evident that an increase in spring damping leads to a slight reduction in the frequencies of Y and Z vibrations, as indicated by the distribution of vibration energy. According to the t detection of the vibration frequency, it is 0.802 and 0.746, both showing |t|<t_0.05 (4)_, indicating that there is no significant difference in the vibration frequency between the two directions. The analysis of variance shows that the significant level of the Y and Z vibration frequencies on different spring damping, P=0.70 and 0.079, are not significant. From **Figure 7**, it can be observed that when the spring damping in three directions increases exponentially, the change in vibration frequency is not significant, especially in the Y direction. Extensive empirical research has established that spring vibration will decay over time, a positive correlation has been observed between the magnitude of spring damping and the duration for vibrations. Therefore, it is advisable to opt for a lower damping value ranging from 0 to 0.3 Ns/mm in order to decelerate the cessation of Y and Z-direction vibrations.

#### 3.1.3 The vibration characteristics on the different blade quality

**Figure 8:**
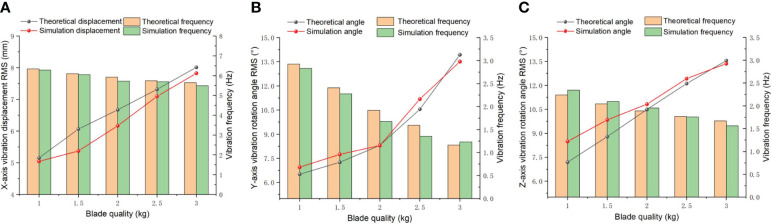
Vibration displacement and variation frequency under different blade quality. **(A)** X direction; **(B)** Y direction; **(C)** Z direction.

The blade is the principal component of the ditching device, which quality influences vibration characteristics. Therefore, the impacts of different blade quality on the vibrations are studied. **Figure 8A** illustrates the relationship between the X-direction vibration displacement and the blade quality under the following conditions: spring stiffness of 10 N/mm in the X direction and 20 N/mm in both the Y and Z directions, along with a spring damping of 0.03 Ns/mm. The weight of the blade can be modified by changing the thickness of the blade. The result reveal a direct correlation between the blade quality and the vibration displacement in all three directions, indicating that as the blade quality increases, the magnitude of vibration displacement also increases correspondingly. When the mass of the blade increases from 1.0 kg to 3.0 kg, the vibration displacement increases from 5.15 mm to 8.01 mm, and the simulated value shows a similar trend to the theoretical value. In the simulation analysis, the RMS value increased from 5.04 mm to 7.82 mm, with an average relative error of 5.17%. The vibration frequency remains between 5.49 and 6.34 Hz. Through ANOVA, it was discovered that the blade quality has a significant impact on the vibration displacement and frequency in the X direction (P<0.01).

The simulated values and the theoretical values exhibit a consistent trend, as depicted in **Figure 8B**. The increase in blade weight led to an escalation in vibration angle displacement along the Y direction, soaring from 6.51° to 13.93°. In the simulation analysis, the RMS value jumped from 6.95° to 13.51°, with an average relative error of 2.43%. **Figure 8C** illustrates that the RMS value of the vibration angle displacement in the Z direction increased from 7.19° to 13.55°, and the simulated value showed a similar trend with the theoretical value. In the simulation analysis, the RMS value increased from 8.49° to 13.36°, with an average relative error of 5.37%. According to the analysis of variance, the significance of the impact of different blade masses on the vibration angular displacement ( ${\text{θ}}_{\text{Y}}$ and ${\text{θ}}_{\text{Z}}$ ) is less than 0.01, indicating a very significant impact effect. A power spectrum analysis on ${\text{θ}}_{\text{Y}}$ and ${\text{θ}}_{\text{Z}}$ is conducted to obtain the spectral characteristics and distribution of vibration energy in the frequency domain. The vibration frequency ${\text{f}}_{\text{Y}}$ decreased from 2.93 Hz to 1.17 Hz and ${\text{f}}_{\text{Z}}$ fallen from 2.24 Hz to 1.67 Hz. In the simulation analysis, the reduction of the vibrations frequency in the Y and Z directions is not significant, because those vibrations originate from the initial disturbance of the spring. The weight of the blade has a direct negative correlation impact on vibration frequency.

As the quality of the blade increases, the vibration displacement and vibration angle both show a linear increase. Excessive vibration displacement and torsion angle usually cause damage to the vibration mechanism and shorten the service life of the device. In addition, the increasing of blades quality has a negative impact on the removal of soil attached to the blades and leads to an increase in resistance between the blades and the soil. Due to the increase in total traction resistance, achieving soil fragmentation becomes more difficult. When the blade mass exceeds 2 kg, a reduction in the vibration frequency is observed, resulting in an escalation in the contact force and area between the soil and blade. Consequently, this factor exerts a discernible impact on the angular displacement of vibrations. To ensure the efficiency of ditching without causing excessive damage to the machinery, it is necessary to avoid an excessive blade vibration angular displacement angle, which can cause unstable vibration of the blade. Therefore, the weight of the blade should be maintained within the range of approximately 1.0 kg under a stable vibration frequency and appropriate vibration angular displacement.

### 3.2 Results and analysis of the average resistance during load ditching

**Figure 9:**
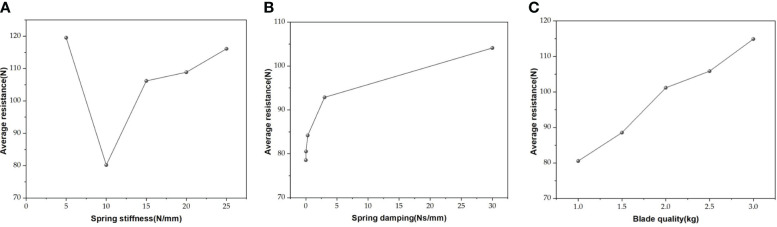
Average resistance of load ditching **(A)** under different spring stiffnesses; **(B)** under different spring damping; **(C)** under different blade qualities.

The magnitude of vibrations in self-excited vibration ditching equipment plays a crucial role in determining both the trajectory of the ditching process and the effectiveness of reducing resistance. According to the significance analysis, the vibration resistance reduction mainly comes from the vibration influence of the X-direction spring stiffness during the ditching process under three different directions of load. Therefore, by using the MBD-DEM coupling simulation model, the variation in the average resistance of load ditching under different spring stiffnesses is explored, as shown in **Figure 9A**. When the stiffness of the X-direction spring is increased, it was observed that the average resistance initially decreases and then increases. At a spring stiffness of 10 N/mm, the average resistance reaches its minimum value of 80.19 N. Furthermore, it was found that the largest reduction in average resistance experienced by the ditching device occurs when the spring stiffness in the X direction ranges from 5 to 10 N/mm. This indicates that the self-excited vibration ditching machine exhibits the most pronounced vibration effect within this range of X-direction spring stiffness. Conversely, when the spring stiffness of X direction is 15-25 N/mm, the average resistance increases linearly. This is because the spring stiffness does not change the X direction vibration frequency, but the displacement of the X-axis vibration gradually decreases when the stiffness spring of the X direction increasing, resulting in a basically unchanged degree of soil vibration fragmentation during the load ditching process. In order to optimize resistance reduction during the load ditching process, it is recommended to maintain the X-direction spring stiffness at approximately 10 N/mm within the self-excited vibration ditching device. From the data depicted in **Figure 9B**, it is clear that the average resistance in vibrations ditching shows a consistent upward trend as the spring damping increases, indicating that the X-direction spring damping has the smallest amplitude of average resistance change within the range of 0 to 0.03 Ns/mm. Due to the change in spring damping does not impact the vibration frequency, resulting in a basically unchanged degree of soil vibration fragmentation during the load ditching process. In conclusion, to optimize resistance reduction during the load ditching process in the ditching device, it is recommended to maintain the X-direction spring damping at approximately 0.03 Ns/mm. The findings demonstrate a linear positive relationship between blade weight and the average resistance encountered during load ditching, as depicted in **Figure 9C**. Due to the increasing of blade quality, the vibration frequency descended remarkably, leading to a decrease in the degree of soil vibration fragmentation during the load ditching process. Additionally, an increase in blade weight effectively mitigates the soil internal stress, thus contributing to a significant rising in average resistance. Overall, to achieve better resistance reduction during the load ditching process, the blade quality should be maintained at approximately 1.0 kg.

### 3.3 Bench test

**Figure 10:**
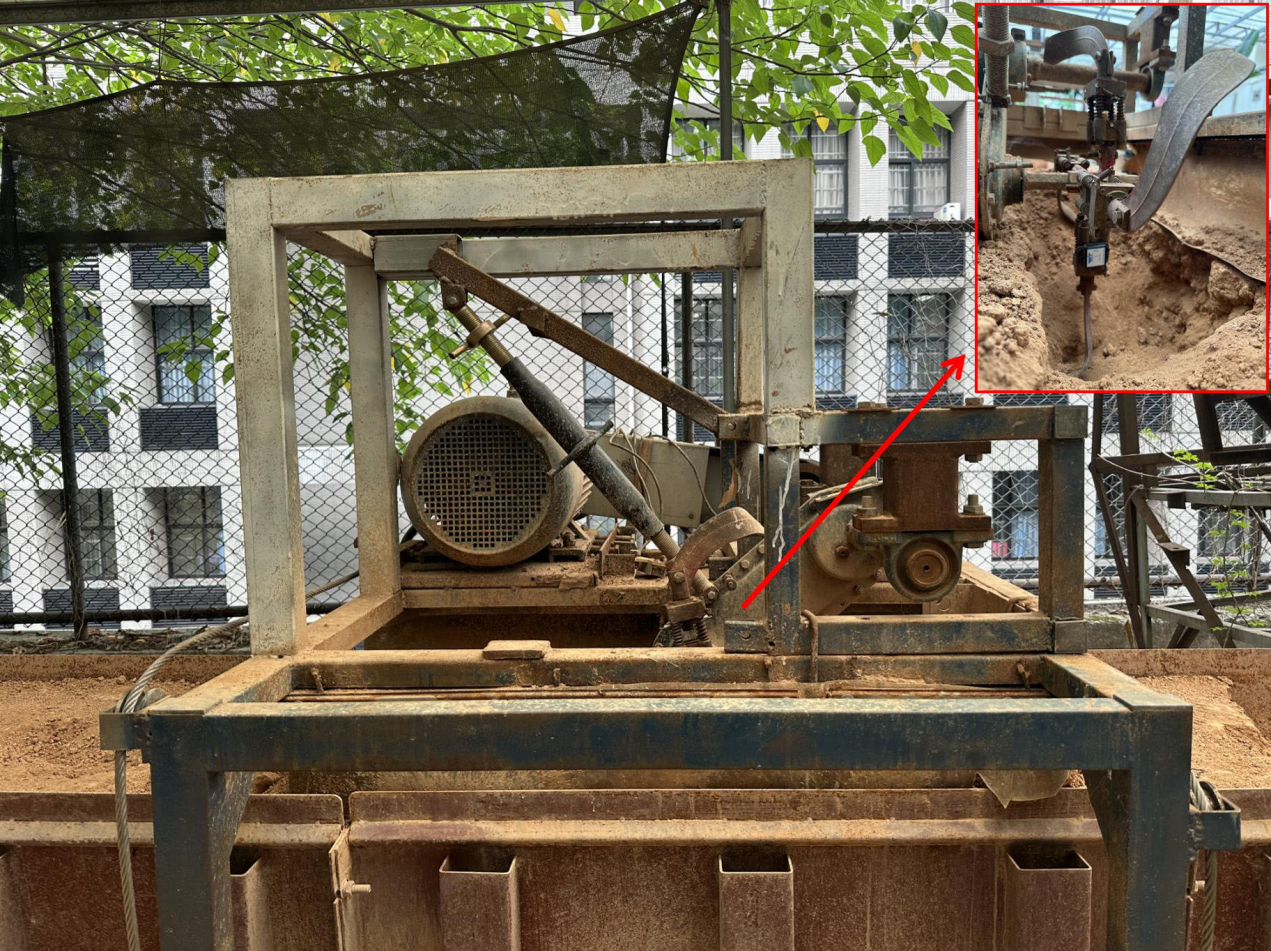
Process of the load ditching average resistance bench test.

Based on the load average resistance using the MBD-DEM coupling model, it can be concluded that the vibration magnitude of the ditching device plays a crucial role in determining the trajectory of the ditching and the extent of resistance reduction achieved by the device. During the load ditching process, the vibration resistance reduction mainly comes from the interaction between the X-direction device and the soil. The vibration of the ditching device in the Y and Z directions is mainly used to avoid hard stones in the soil. **Figure 10** illustrates the load ditching test bench, where suitable structural parameters have been selected. The X-direction spring dimensions are chosen as 1.6 mm (wire diameter) × 13 mm (diameter) × 35 mm (length), with a corresponding spring stiffness of 9.9 N/m. The spring damping is set at 0.03 N/s/m, and the mass of the blade is determined as 1 kg. The ditching device was set to a forward speed of 0.3 m/s, while the blade rotated in a counterclockwise direction. Shaikh et al. (2021) stated that the maximum traction force occurs at the height of 45 mm soil layer with the moisture of 21.5%. In order to ensure parameter consistency throughout each experiment, a moisture content measuring instrument was utilized to measure the soil moisture content, within the range of 15% to 17%. This moisture content is considered a key parameter when ditching specific soil types during fertilization process (Wang J. F., et al., 2023). Additionally, the TYD-2 soil hardness tester was utilized to measure the soil compaction, yielding an approximate value of 355 KPa. According to horticultural requirements, adjust the ditching depth to 30cm consistent with the simulation parameter, and use the ditching depth adjustment support frame of the test bench for adjustment.

**Figure 11:**
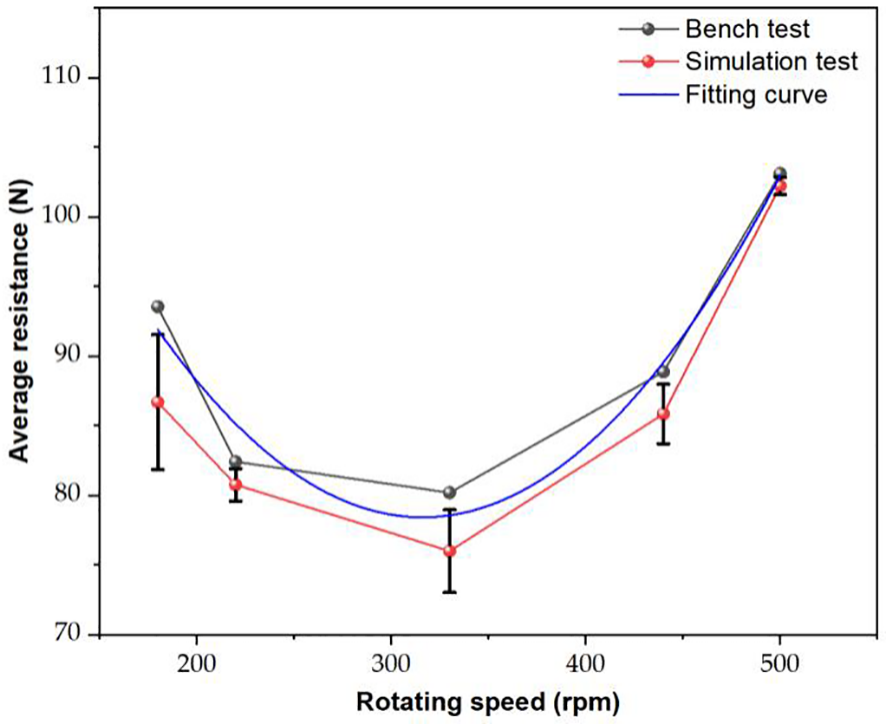
The average resistances of the bench test and MBD-DEM coupling simulation.

Through MBD-DEM coupling simulation and load ditching bench tests, the variations in the average load resistances of ditching device under different rotating speeds were analyzed. The results is shown in **Figure 11**, showing that the average resistance first decreases and then increases with the rotating speed rising, reaching the lowest resistance of 80.19 N at 330 rpm. The trend of variation between the simulated and experimental values is basically the same. At a rotational speed of 330 rpm, the average resistance reaches its minimum value of 76 N, exhibiting an average relative error of 5.23%. Wang et al. (2020) proposed a vibrating subsoiler applied in DEM simulation model, which error of the tillage resistance is less than 9.65%, indicating that the coupling simulation model can more accurately simulate the interaction between vibration device and soil.

The average resistance values obtained from the bench test are generally higher than the values obtained from the coupling simulation. This difference can be attributed to various factors, including power consumption due to obstacles such as stones in the soil, friction in the transmission system, and the forward resistance. By fitting the data pairs, the following fitting equation can be obtained Equation 9:


(9) $$\text{y}=7.26\times 10^{-4}{\text{x}}^{2}-0.46\text{x}+150.00,\;{\text{R}}^{2}=0.96\;$$

This indicates that the MBD-DEM coupling model results can better reflect the changes in the ditching resistances. Hence, it can be concluded that the coupling simulation model established in this study is reliable and can be effectively utilized to provide the resistance reduction effect of ditching blade. The resistance parameters of fixed degrees and releasing degree of freedom ditching blades were measured under the conditions of setting parameters for ditching depth and width to 30cm and 15cm. The test results were obtained by taking the average value of three sets of tests. A comparison between two different blades and the reference condition reveals the depth and width of the ditching, as presented in **Table 3**.

**Table 3:** Ditching depth and width of non-self-excited and self-excited vibration ditching.

	Non-self-excited vibration ditching		Self-excited vibration ditching	
	Depth (cm)	Width (cm)	Depth (cm)	Width (cm)
180r/min	30	15.2	29.6	16
220r/min	29.7	15.1	30.1	16.3
330r/min	29.6	15.3	29.9	16.5
440r/min	29.6	15.9	29.72	16.2
500r/min	29.86	15.6	29.1	15.9

**Figure 12:**
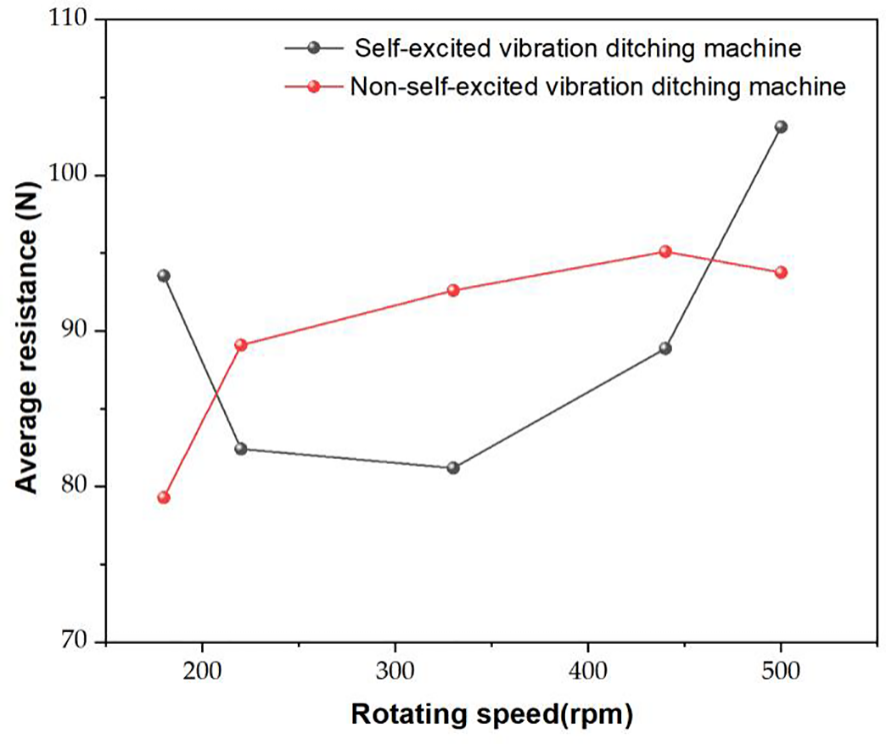
Comparison results of average resistance test.

The comparison results, as depicted in **Figure 12**, reveal that the variation in ditch depth and width is within 10% of the set value under the test conditions for both the ditching blades. Because in practical operation, the soil has a certain degree of looseness, and the plow or blade of the ditching machine will apply a certain amount of pressure and shear force to the soil, resulting in the actual width of the ditching being larger than the set value. Within the speed range of 220-440 rpm, the self-excited vibration ditching device exhibits an obvious reduction in average resistance compared to the non-vibration method. The maximum reduction observed is 12.3%, indicating a significant improvement in resistance reduction during the ditching process. Zhou et al. (2019) developed a sliding cut type self-excited vibration resistance reduction deep loosening device with inner and outer double springs, which can reduce resistance by 15.45%~20.05% compared to traditional deep loosening shovels. However, the ditching method without self-excited vibration performs better under low and high RPMs (180 rpm and 500 rpm) operating conditions. The self-excited vibration ditching method has a higher average resistance than the non-self-excited vibration ditching method. This indicates that under low and high-speed operating conditions, the soil breaking force of the self-excited vibration ditching blade varies sharply. However, within the medium-speed range, the fluctuation of the soil breaking force is smaller than that of the traditional ditching blade.

## 4 Conclusion

In summary, this article proposes a self-excited vibration ditching device. A simulation model of the MBD-DEM coupled system was established considering the characteristics of the soil and the interaction between the soil and the ditching blade. The effects of vibration characteristics on tillage resistance and soil disturbance was explored, and the following key conclusions were drawn:

(1) The single factor experiment shows that the average resistance of the ditching device exhibits a non-linear trend with the increase of spring stiffness, initially decreasing and then increasing. However, with the improvement of spring damping and blade quality, the average resistance experienced by the device shows an upward trend.
(2) The results of the load bench test show that the average resistance variation trend between the simulated and experimental values is similar, with an average relative error of 5.23%, verifying the high accuracy of the MBD-DEM coupling simulation model.
(3) The kinematic analysis results indicate that the vibrating ditching device has better soil crushing effect. Compared with traditional vibration ditching blade, the average resistance can be reduced by 12.3% at a speed of 330r/min, showing a significant resistance reduction effect.

The proposed coupling simulation method can simulate the dynamic behavior of complex systems, reduce the development cost of agricultural machinery. In future research, to further reduce ditching resistance and predict the durability and reliability of device under actual working conditions, the MBD-DEM coupling simulation model will be optimized. Specifically, the optimization of agricultural machinery and the design of device components will be increasingly in the focus and coupling simulation models will simulate wear and energy consumption during operation. An effective simulation model can guide the structure design of components and the selection of material, extending the service life of agricultural machinery.

## Data availability statement

The raw data supporting the conclusions of this article will be made available by the authors, without undue reservation.

## Author contributions

YZ: Methodology, Resources, Software, Writing – original draft, Writing – review & editing, Formal analysis, Project administration. JL: Funding acquisition, Supervision, Writing – review & editing, Writing – original draft. HL: Data curation, Resources, Software, Writing – original draft. QZ: Conceptualization, Methodology, Resources, Writing – original draft. CL: Software, Visualization, Writing – original draft. ZL: Conceptualization, Data curation, Investigation, Writing – original draft. RJ: Investigation, Resources, Validation, Writing – original draft. CM: Investigation, Project administration, Visualization, Writing – original draft. ZM: Funding acquisition, Project administration, Resources, Writing – original draft. HH: Resources, Validation, Visualization, Writing – original draft.
